# Is it Possible To Identify Patients After Their First Hospitalization for a Psychotic Disorder Who Do Not Use Anti-Psychotics and are Not Later Rehospitalized?

**DOI:** 10.1093/schbul/sbaf011

**Published:** 2025-02-21

**Authors:** Amir Krivoy, Jari Tiihonen, Johnatan Nissan, Arad Dotan, Dana Arnheim, Noa Menkes-Caspi, Sharon Taub, Heli Tuppurainen, Ellenor Mittendorfer-Rutz, Michael Davidson, John M Davis, Mark Weiser, Heidi Taipale

**Affiliations:** Geha Mental Health Center, 49100 Petach-Tikva, Israel; Mental Health Data Research Center, Clalit Health Services, 49100 Petach-Tikva, Israel; School of Medicine, The Faculty of Medical and Health Sciences, Tel-Aviv University, 39040 Tel-Aviv, Israel; The Department of Clinical Neuroscience, Karolinska Institutet, 171 77 Stockholm, Sweden; Center for Psychiatry Research, Stockholm City Council, 171 77 Stockholm, Sweden; Department of Forensic Psychiatry, University of Eastern Finland, FIN-70240 Kuopio, Finland; Niuvanniemi Hospital, FIN-70240 Kuopio, Finland; School of Medicine, The Faculty of Medical and Health Sciences, Tel-Aviv University, 39040 Tel-Aviv, Israel; Zachai Division of Psychiatry, Sheba Medical Center, 52621 Ramat-Gan, Israel; School of Medicine, The Faculty of Medical and Health Sciences, Tel-Aviv University, 39040 Tel-Aviv, Israel; Zachai Division of Psychiatry, Sheba Medical Center, 52621 Ramat-Gan, Israel; School of Medicine, The Faculty of Medical and Health Sciences, Tel-Aviv University, 39040 Tel-Aviv, Israel; Zachai Division of Psychiatry, Sheba Medical Center, 52621 Ramat-Gan, Israel; Geha Mental Health Center, 49100 Petach-Tikva, Israel; Mental Health Data Research Center, Clalit Health Services, 49100 Petach-Tikva, Israel; Geha Mental Health Center, 49100 Petach-Tikva, Israel; Mental Health Data Research Center, Clalit Health Services, 49100 Petach-Tikva, Israel; Department of Forensic Psychiatry, University of Eastern Finland, FIN-70240 Kuopio, Finland; Niuvanniemi Hospital, FIN-70240 Kuopio, Finland; The Department of Clinical Neuroscience, Karolinska Institutet, 171 77 Stockholm, Sweden; Department of Basic and Clinical Sciences, University of Nicosia Medical School, 2408 Nicosia, Cyprus; University of Illinois, Chicago, IL 1200, United States; School of Medicine, The Faculty of Medical and Health Sciences, Tel-Aviv University, 39040 Tel-Aviv, Israel; Zachai Division of Psychiatry, Sheba Medical Center, 52621 Ramat-Gan, Israel; The Department of Clinical Neuroscience, Karolinska Institutet, 171 77 Stockholm, Sweden; Department of Forensic Psychiatry, University of Eastern Finland, FIN-70240 Kuopio, Finland; Niuvanniemi Hospital, FIN-70240 Kuopio, Finland; School of Pharmacy, University of Eastern Finland, FIN-70240 Kuopio, Finland

**Keywords:** psychotic disorders, anti-psychotics, relapse, discontinuing, prediction

## Abstract

**Background:**

Guidelines issued by professional organizations recommend that all patients with psychotic disorders who have had several psychotic relapses, continue maintenance anti-psychotic treatment. However, some patients discontinue anti-psychotics and do not later relapse. This study attempted to characterize those patients with psychotic disorders early in their disease not taking maintenance antipsychotics, who were not later hospitalized.

**Study Design:**

This population-based cohort study combined registry data on patients diagnosed in their first psychotic episode (ICD 10 code: F20-29) from Sweden (*n* = 20 848), and Israel (*n* = 10 045), and followed them for up to 7 years for re-hospitalization or death. Multivariate analyses assessed sociodemographic and clinical risk factors predicting rehospitalization or death in patients with one hospitalization and did not fill prescriptions for antipsychotics; results from Sweden and Israel were then meta-analyzed.

**Study Results:**

The main analysis of this paper included 1611 patients from Sweden and 1607 from Israel. Male gender (adjusted hazard ratio [aHR], 1.57; 95% confidence interval [CI], 1.16-2.13) and a diagnosis of narrowly defined schizophrenia (F20.0-F20.9; aHR, 1.85; 95% CI, 1.55-2.2) were associated with increased risk of a second hospitalization or death among those who did not use antipsychotics. No sociodemographic or clinical characteristics were associated with a decreased risk of a second hospitalization or death.

**Conclusions:**

Based on registry data, it was not possible to characterize, in a clinically meaningful way, those patients who can safely discontinue anti-psychotic medications and not be re-hospitalized or die. Male gender and a diagnosis of narrowly defined schizophrenia were associated with an increased risk of later relapse.

## Introduction

Before antipsychotic drugs were discovered there were a minority of patients (20–35%) who had a single psychotic episode and never had a relapse, this is reflected in the ICD-10 (International Classification of Diseases) diagnosis of “acute and transient psychotic disorders” and DSM 5 (Diagnostic and Statistical Manual of Mental Disorders) diagnosis of “Brief Psychotic Disorder.”^1^ More recent follow-up studies of patients treated psychoanalytically without antipsychotics, studies of non-compliant patients, and various other follow-up studies reported that some drug-free patients do not relapse.^2–6^ Many randomized controlled trials (RCTs) and meta-analyses find that the majority of patients who do not take antipsychotic medications do relapse.^7,8^ However, most of these studies were funded by the companies who manufacture these medications, for registration purposes, on patients at high risk for relapse. Hence, these trials may not reflect the full range of psychotic disorders. Based on those data, most treatment guidelines recommend that individuals who meet diagnostic criteria for schizophrenia and other chronic psychotic disorders and experience recurrent psychotic relapses should continue maintenance antipsychotic treatment indefinitely.^9–13^ However, many patients do not take antipsychotic medications, in part due to side effects, stigma, or lack of insight into the need for the treatment.^14^ As clinicians often advise their patients to continue maintenance treatment, this lack of agreement is often a cause of patient discontent ending in discontinuation, often leading to relapse and sometimes hospitalization.

That being said, some of the patients who discontinue antipsychotic treatment do not later relapse, suggesting that they do not need to be treated with long-term antipsychotics. Hence, it would be ideal to identify those patients who can safely discontinue antipsychotics without risk of relapse, and recommend them to discontinue, while advising those patients who cannot safely discontinue anti-psychotics, not to do so. This approach might be perceived as a “personalized medicine” approach which might be attractive to both clinicians and patients.

A review on the topic^15^ found that older age, later onset of illness, shorter duration of untreated psychosis, modest psychopathology, better social functioning, and lower doses of antipsychotics were associated with a lower risk of relapse.

The purpose of this current study was to study patients with psychotic disorders from 2 countries from different continents early in their illness and follow them up for up to 7 years using real-world data, in an attempt to characterize those patients who can safely discontinue antipsychotic treatment and do not relapse. Characterizing these patients might enable clinicians to make evidence-based recommendations for patients who can safely discontinue antipsychotic treatment. The primary analysis in this study is on patients with one hospitalization who discontinued antipsychotic medications and is later hospitalized. This paper also examines the risk for relapse in patients with psychotic disorders other than schizophrenia.

## Methods

This study combined population-based cohort data from Sweden and Israel. As clinical data for relapse are not available to us, the data in this study were extracted from hospitalization registries, and hospitalization or death are used as proxies for relapse. However, rehospitalization and relapse are not always synonymous; some patients relapse but are not rehospitalized, and others are rehospitalized but do not relapse.

### Ethics

This project was approved by the Regional Ethical Review Board, Karolinska Institutet, Stockholm, Sweden (Dnr 2007/762-31 and Dnr 2021-06441-02), by the Institutional Review Boards (IRB) of the Geha Mental Health Center and the Data Committee of the Clalit Health Services, and by the IRB of the Sheba Medical Center.

### Study Design

#### Swedish Cohort

The Swedish study cohort was identified from nationwide registers, specifically the National Patient Register, consisting of data on all inpatient stays and outpatient visits, and the Microdata for Analyses of Social Insurance (MiDAS) register, which includes sickness absences and disability pensions. Using a pseudonymized personal identification number, these data were then linked to other national registries, including the Prescribed Drug Registry, with data on all filled prescriptions from Swedish pharmacies. Sociodemographic variables were obtained from the Longitudinal Integration Database for Health Insurance and Labour Market Studies.

The Swedish cohort included all patients first diagnosed with a non-affective psychotic spectrum disorder (ICD10 F20-F29) between 2006 and 2019, aged 16–41 at diagnosis, without a previous diagnosis of schizophrenia-spectrum disorder before 2006 in any of the registers used. All medications were coded using the ATC (Anatomic Therapeutic Chemical) classification system.^16^

#### Israeli Cohort

The study utilized population-based data from the Clalit Health Services registry, the largest health maintenance organization (HMO) in Israel (*n* = 4.8 million, 53% of the population). This electronic database was established in the year 2000 and is updated and validated using multiple sources of diagnoses (medical facilities, administrative operating systems, and pharmacies). The database includes diagnoses of psychotic disorders made in psychiatric hospitals as well as diagnoses recorded by general practitioners in the outpatient medical files, based on diagnoses made by psychiatrists. The diagnosis of schizophrenia was validated in a previous study, finding 94% to be accurate.^17^ Data on all filled prescriptions were extracted from the pharmacy registry.

The Israeli cohort included patients first diagnosed with a non-affective psychotic spectrum disorder (ICD10 F20-F29) between July 1, 2015 and June 30, 2022, aged 16-41 at diagnosis, without a previous diagnosis of schizophrenia-spectrum disorder before July 1, 2015, in the HMO registry. All medications were coded using the ATC classification system.

### Inclusion and Exclusion Criteria

Patients diagnosed for the first time with a non-affective psychotic disorder between the ages of 16 and 41 at diagnosis, with at least 2 years of follow-up in the registries were included. Patients who used antipsychotics for 15 months before the first diagnosis was made were excluded. This age range includes the majority of patients in their first psychotic episode; patients hospitalized for the first time after age 41 were not included because they are not representative of the majority of patients with a psychotic disorder, are more likely to be female, have less severe positive symptoms, and need lower doses of antipsychotics.^18^ Data on patients under age 16 were not available to us. A limit of a maximum of 180 days for hospitalizations was chosen to exclude patients with very long hospital care periods (due to for example, forensic psychiatric care). This limit was needed to exclude outliers who could not be considered as “true” incident cases when they are discharged from their first hospitalization, which could last up to 7 years. At that point, they are not what is generally considered as “first-episode psychosis” patients as they actually have a very long duration of illness. In the Swedish cohort, 2.8% had longer than 180 days of first hospitalization, and in the Israeli cohort, it was 5.5% of the patients.

### Socio-Demographic and Clinical Variables

The study cohorts were stratified according to where the first diagnosis was assigned (inpatient vs outpatient). Those patients initially diagnosed with a psychotic disorder as outpatients but who were hospitalized within 30 days after their first outpatient diagnosis were considered as having received their first diagnosis as inpatients.

The follow-up period started at the date of the first diagnosis (or discharge for inpatients), and ended at death, after 7 years, or at the date of the data linkage (December 31, 2021, for the Swedish data, June 30, 2022, in for the Israeli data). The Swedish data also included emigration as a cause of end of follow-up, data on emigration were not available for the Israeli cohort.

As the focus of this study was on patients with a chronic psychotic disorder early in their illness, the main analyses focused on patients hospitalized once and did not fill prescriptions for antipsychotic medication after discharge. The follow-up for the cohort with one hospitalization started at discharge from the first hospitalization and ended at a second hospitalization due to psychosis, death, or the end of follow-up period, that ever came first. Because the diagnosis of patients after one admission might not be well established, we also conducted analyses on patients with 2 hospitalizations, and their risk for a third hospitalization or death.

We assessed the variables available in the databases as potential predictors of relapse. ICD-10 psychotic disorder diagnoses were dichotomized, either F20 (schizophrenia), or “other”: F21 (schizotypal disorder), F22 (persistent delusional disorders), F23 (acute transient psychotic disorder), F24 (induced delusional disorder), F25 (schizoaffective disorder), F28 (other non-organic psychotic disorder), and F29 (unspecified psychosis). Age, sex, socioeconomic status (SES; low, medium, high), substance use disorder (F10-F19 recorded ever or within 3 months after diagnosis), and use of benzodiazepines (ATC N05BA, N05CD, N05CF) or antidepressants (N06A) were also recorded. Use of these medications was measured from 30 days before to 30 days after the first hospitalization. The SES variable in the Swedish cohort was based on education (<9 years = low, 9–11 years = medium, ≥12 years = high), and in the Israeli cohort was based on neighborhood socioeconomic status.^19^

### Statistical Analyses

The focus of this report is an attempt to characterize those patients with a psychotic disorder early in their illness who did not take antipsychotic medications and were not later hospitalized.

Among patients who did not use antipsychotics, risk for a second hospitalization or death was calculated for patients after their first hospitalization; for patients with 2 hospitalizations analyses assessed risk for a third hospitalization or death. The analyses were conducted separately on the Swedish and Israeli cohorts, the adjusted results were meta-analyzed (random effects meta-analysis) with R Studio, metafor package (version 3.0-2). The Swedish data were analyzed using SAS 9.4; the Israeli data were analyzed using Python. The results are reported as unadjusted and adjusted hazard ratios (aHRs) with 95% confidence intervals (CIs).

Separate analyses on the risk for a second hospitalization in patients diagnosed with each ICD 10 psychotic diagnosis (F21-F29) in their first hospitalization were also calculated.

## Results

### Sweden

The study included 20 549 first-episode patients from Sweden. Approximately half were initially diagnosed as outpatients (49.9%, *N* = 10 253) and half as inpatients (50.1%, *N* = 10 296). Of those initially diagnosed as outpatients, 2566 (25.0%) did not fill prescriptions for antipsychotics, with a relapse rate of 7.7%. Of the 11 816 patients hospitalized for their first time (regardless of whether they were initially diagnosed as outpatients or inpatients), 1611 (13.6%) did not fill prescriptions for antipsychotics, with a relapse rate of 23.7%. Of the patients hospitalized for the first time, 4485 were hospitalized twice. Of those who were hospitalized twice, 463 (7.6%) did not fill prescriptions for antipsychotics, with a relapse rate of 43.6% (Figure S1A).

### Israel

There were 10 045 first-episode patients from Israel, 6095 (60.7%) were diagnosed as outpatients and 3950 (39.3%) were diagnosed as inpatients. Of those initially diagnosed as outpatients, 3329 (62.8%) did not fill prescriptions for antipsychotics, with a relapse rate of 12.5%. Of the 4470 patients hospitalized for the first time (regardless of whether they were initially diagnosed as outpatients or inpatients), 1607 (36%) did not fill prescriptions for antipsychotics, with a relapse rate of 52%. Of the patients hospitalized for the first time, 1704 were hospitalized twice. Of those who were hospitalized twice, 535 (31.4%) did not fill prescriptions for antipsychotics, with a relapse rate of 72.1% (Figure S1B).

Table 1 shows the characteristics of the Israeli and Swedish cohorts with one hospitalization and not using antipsychotics. The mean age of the patients in the Swedish cohort was 28.1 ± 6.6, of whom 30.7% (*N* = 495) were females. The mean age of the patients in the Israeli cohort was 27.3 ± 6.7, of whom 33.2% were females; more patients in the Swedish cohort had low SES. The diagnosis of schizophrenia was less frequent in the Swedish cohort than in the Israeli cohort (4.0% vs 43.3%, respectively), whereas other psychotic disorders, especially unspecified non-organic psychotic disorder, were more commonly recorded in the Swedish cohort (37.2% vs 18.8%, respectively).

**Table 1.** Characteristics of Israeli and Swedish Study Cohorts of Patients With Psychotic Disorders Who Did Not Use Antipsychotics After the First Hospitalization

	Israeli cohort *N* = 1607	Swedish cohort *N* = 1611
Mean age ± SD	27.3 ± 6.7	28.1 ± 6.6
Female sex	33.2% (533)	30.7% (495)
Diagnosis type at second hospitalization		
Schizophrenia	43.3% (696)	4.0% (65)
Acute and transient psychotic disorder	29.4% (472)	47.4% (763)
Unspecified psychosis	18.8% (302)	37.2% (600)
Other	8.5% (137)	11.4% (183)
Socioeconomic position^a^		
Low	17.2% (277)	48.4% (780)
Medium	60.0% (965)	35.8% (577)
High	17.7% (285)	15.8% (254)
Parental education^b^	NA	
Unknown	NA	27.5 (443)
Low	NA	12.7 (204)
Medium	NA	32.2 (519)
High	NA	27.6 (445)
Substance use disorder	27.8% (446)	43.5% (701)
Benzodiazepine use	10.4% (167)	23.4% (377)
Antidepressant use	9.3% (149)	29.2% (470)
Disability pension	NA	8.6% (139)
Second hospitalization due to psychosis	50.2% (807)	16.7% (269)
Death during follow-up	2.3% (42)	7.8% (125)

^a^Refers to educational level in the Swedish data and socioeconomic position in Israeli data. *N* = 80 patients had missing data regarding their socioeconomic position at the Israeli data.

^b^Parental education is only available for the Swedish data. Unknown educational level mainly refers to people whose parents are not residents in Sweden, or for whom educational level is not recorded (e.g., education not completed in Sweden).

Table 2 shows country-specific risks of relapse controlling for sociodemographic and clinical factors. Unadjusted analyses of the associations between sociodemographic and clinical characteristics and the risk of a second hospitalization or death showed that older age (≥30 years), male gender, diagnoses of narrowly defined schizophrenia, and substance use disorder (SUD) were associated with increased risk of rehospitalization or death in the Swedish cohort, while high socioeconomic status was associated with a decreased risk. In the Israeli cohort male gender, diagnoses of narrowly defined schizophrenia, SUD, benzodiazepine use, and antidepressant use were associated with increased risk, while high socioeconomic status was associated with a decreased risk. In the adjusted analyses, older age, male gender, diagnoses of narrowly defined schizophrenia, and SUD were associated with an increased risk of second hospitalization or death in the Swedish data. In the Israeli data, SUD, benzodiazepine use, antidepressant use, and diagnoses of narrowly defined were associated with increased risk of second hospitalization or death.

**Table 2.** Risk of Second Hospitalization Associated With Sociodemographic and Clinical Characteristics in Israeli and Swedish Cohorts of Persons With First-Episode Schizophrenia-Spectrum Disorder After First Hospitalization

Israeli cohort					Swedish cohort			
No relapse		Relapse	Crude	Multivariate	No relapse	Relapse	Crude	Multivariate
	*N* = 772	*N* = 835	HR (95% CI)	HR (95% CI)	*N* = 1230	*N* = 381	HR (95% CI)	HR (95% CI)
Age, categorized, % (*N*)								
<30 years	47.2% (497)	52.8% (556)	1.07 (0.93-1.24)	1.15 (0.99-1.33)	78.8% (731)	21.2% (197)	0.76 (0.62-0.93)	0.72 (0.59-0.89)
≥ 30 years	49.6% (275)	50.4% (279)	ref	ref	73.1% (499)	26.9% (184)	ref	ref
Male	44.4% (447)	55.4% (597)	1.35 (1.16-1.57)	1.12 (0.95-1.31)	72.9% (813)	27.2% (303)	1.92 (1.49-2.47)	1.85 (1.43-2.40)
Diagnosis type at 1st hospitalization								
F20 Schizophrenia	42.5% (296)	57.5% (400)	1.31 (1.15-1.5)	1.36 (1.18-1.56)	56.9% (37)	43.1% (28)	1.91 (1.30-2.81)	2.09 (1.42-3.09)
Other	52.2% (476)	47.8% (435)	Ref	Ref	77.2% (1193)	22.8% (353)	Ref	Ref
SES								
Low	54.7% (156)	45.3% (129)	1.01 (084-1.21)	0.88 (0.73-1.05)	75.5% (589)	24.5% (191)	0.98 (0.79-1.22)	1.05 (0.84-1.31)
Medium	46.2% (446)	53.8% (519)	Ref	Ref	75.2% (434)	24.8% (143)	Ref	Ref
High	48.4% (134)	51.6% (143)	0.77 (0.64-0.93)	0.86 (0.71-1.04)	81.5% (207)	18.5% (47)	0.69 (0.50-0.97)	0.81 (0.58-1.14)
No data	45% (36)	55% (44)						
Substance use disorder	27.6% (123)	72.4% (323)	2.13 (1.86-2.46)	2.01 (1.74-2.33)	72.0% (505)	28.0% (196)	1.45 (1.18-1.77)	1.23 (1.01-1.53)
Benzodiazepine use	40.27% (60)	59.7% (89)	1.69 (1.39-2.06)	1.5 (1.22-1.85)	74.0% (279)	26.0% (98)	1.19 (0.95-1.50)	1.11 (0.84-1.46)
Antidepressant use	30.5% (51)	69.5% (116)	1.31 (1.05-1.64)	1.29 (1.02-1.63)	75.1% (353)	24.9% (117)	1.14 (0.92-1.42)	1.09 (0.84-1.42)

**Figure 1.**
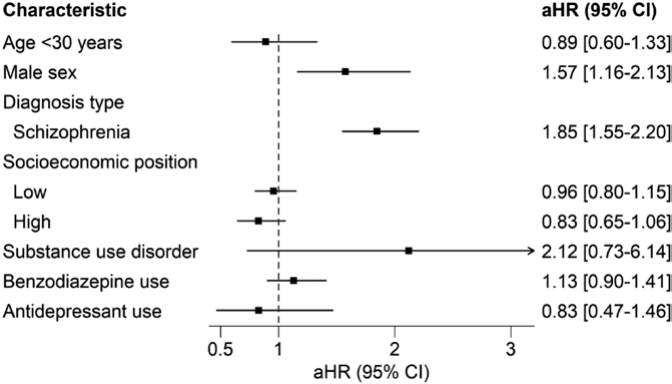
Meta-Analysis Results on the Risk of Second Hospitalization Associated With Sociodemographic and Clinical Characteristics in Israeli and Swedish Cohorts of Persons With First-Episode Schizophrenia-Spectrum Disorder Diagnosed After First Hospitalization

Figure 1 shows the results of the meta-analyzed results combining the 2 datasets. Male gender (adjusted hazard ratio [aHR], 1.57; 95% CI, 1.16-2.13), and diagnoses of narrowly defined schizophrenia (aHR, 1.85; 95% CI, 1.55-2.2) were associated with increased risk for a second hospitalization or death. No clinical or demographic variable was identified as protective against second hospitalization or death.

The focus of this paper was on patients who did not use antipsychotics after their first hospitalization.

**Figure 2.**
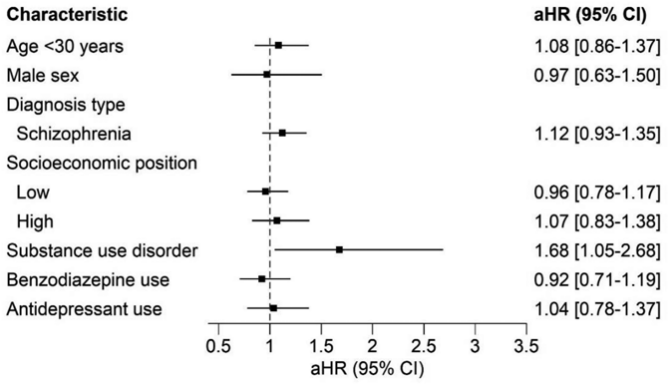
Random Effects Meta-Analysis of Israeli and Swedish Patients with Psychotic Disorders, After Discharge From Their Second Hospitalization, Who Do Not Take Antipsychotics, Who Did or Did Not Have a Third Hospitalization. Adjusted Hazard Ratios with 95% Confidence Intervals

The risk of a third hospitalization or death for patients hospitalized twice who did not take antipsychotics is shown in Table 3. In the Swedish dataset, no variables were significantly associated with an increased risk for a third hospitalization, in the Israeli dataset only SUD was associated with an increased risk. The meta-analysis combining the Swedish and Israeli cohorts found that only substance use disorder was associated with an increased risk for a third hospitalization or death (Figure 2).

**Table 3.** Sociodemographic and Clinical Characteristics of Swedish and Israeli Patients With Psychotic Disorders After Discharge From Their Second Hospitalization, Who Do Not Take Antipsychotics, Who Did or Did Not Have A Third Hospitalization. Hazard ratios (HRs) With 95% Confidence Intervals (CIs)

Israeli cohort					Swedish cohort			
No third hospitalization		Third hospitalization	Unadjusted	Adjusted	No third hospitalization	Third hospitalization	Unadjusted	Adjusted
	*N* = 149	*N* = 386	HR (95%CI)	HR (95%CI)	*N* = 261	*N* = 202	HR (95%CI)	HR (95%CI)
Age, categorized, % (*N*)								
<30 years	29.2% (92)	70.7% (222)	0.91 (0.74-1.11)	0.98 (0.79-1.20)	50.8% (94)	49.2% (91)	1.33 (1.00-1.75)	1.25 (0.93-1.68)
≥30 years	25.8% (57)	74.2% (164)	ref	ref	64.0 (167)	39.9% (111)	ref	ref
Male	27.7% (106)	72.3% (277)	0.98 (0.79-1.23)	0.79 (0.62–1.00)	53.8% (174)	46.1% (149)	1.35 (0.99-1.85)	1.24 (0.84-1.83)
Diagnosis type at second hospitalization								
Schizophrenia	30.9% (90)	69.1% (201)	1.02 (0.83-1.24)	1.09 (0.88-1.34)	54.6% (33)	48.4% (31)	1.22 (0.83-1.78)	1.24 (0.84-1.83)
Other	24.2% (59)	75.8% (185)	ref	ref	57.1% (228)	42.9% (171)	ref	ref
Socioeconomic position^a^								
Low	33.3% (26)	66.7% (52)	1.01 (0.79-1.30)	0.90 (0.69-1.16)	54% (115)	46% (98)	1.18 (0.87-1.60)	1.05 (0.77-1.44)
Medium	26.8% (86)	73.1% (234)	ref	ref	58% (98)	42% (71)	ref	ref
High	25.5% (26)	74.5% (76)	0.88 (0.66-1.18)	1.04 (0.76-1.42)	59.3% (48)	40.7% (33)	0.97 (0.64-1.47)	1.13 (0.74-1.74)
Substance use disorder	13% (31)	87% (208)	1.97 (1.61-2.41)	2.11 (1.70-2.63)	52.3% (111)	47.6% (101)	1.36 (1.03-1.80)	1.31 (0.97-1.76)
Benzodiazepine use	26.2% (22)	73.8% (62)	1.03 (0.79-1.35)	1.00 (0.75-1.33)	63.3% (38)	36.7% (22)	0.72 (0.46-1.12)	0.75 (0.46-1.20)
Antidepressant use	33.3% (19)	66.7% (38)	0.93 (0.67-1.30)	0.97 (0.68-1.38)	55.6% (30)	44.4% (24)	0.99 (0.65-1.51)	1.16 (0.74-1.83)

^*^Thirty-five patients had missing data regarding their socioeconomic position in the Israeli data.

Table S2 shows the association between the various psychotic disorders and risks of a second hospitalization in patients hospitalized once and not using antipsychotics.

Figures S1A and S1B show the relapse rates of all patients in the early stages of illness, both did and did not take antipsychotics. Table S3 shows the association between parental educational level and risk of first hospitalization in the Swedish cohort.

## Discussion

The main purpose of this study was to investigate if it is possible to characterize those patients with psychotic disorders in the first years of illness who can safely discontinue antipsychotic treatment without later being hospitalized. This might enable a personalized medicine approach, recommending maintenance treatment only for those patients who need it, and not for all patients with psychotic disorders with recurrent hospitalizations. Unfortunately, using these registry data, it was not possible to identify these patients. We did find that male patients and those diagnosed with narrowly defined schizophrenia (F20) are at increased risk for later hospitalizations or death. However, these characteristics are not specific and do not enable accurate identification of patients who can safely discontinue antipsychotic treatment.

These data expand on the current literature on this topic. A recent review^15^ on the clinical characteristics of patients with schizophrenia who successfully discontinued antipsychotic treatment evaluated RCTs and naturalistic studies. Some of these studies were relatively short, and none population-based. The authors of that review found that older age, later onset of illness, shorter duration of untreated psychosis, modest psychopathology, better social functioning, and being maintained on a lower dose of antipsychotics before discontinuation were associated with a lower risk of relapse. Another systematic review and meta-analysis on this topic attempted to identify predictors of successful discontinuation of anti-psychotics in patients after their first psychotic episode. No clinical characteristics correctly predicting successful anti-psychotics discontinuation were identifed.^20^

The topic of whether patients with schizophrenia can safely discontinue antipsychotic treatment has been a subject of several recent RCTs, finding that discontinuation or dose reduction of antipsychotic medications was associated with an increased risk of later hospitalizations. Wunderink et al. performed a gradual reduction of antipsychotic dose and discontinuation of antipsychotics in people with a first episode of psychosis. In that trial, at the 18-month follow-up, there was an increased rate of relapse in participants allocated to antipsychotic reduction.^21^ The recent RADAR trial^22^ examined gradual antipsychotic medication dose reduction and discontinuation and also reported increased rates of relapse in patients with dose reduction. These papers did not specify the characteristics of patients who could successfully discontinue treatment without relapse.

We found similar findings, which emphasize the clinical importance of encouraging patients to take antipsychotics and closely follow patients who do not take antipsychotics. However, this might be challenging as many of the patients who do not take antipsychotics are not interested in psychotic follow-up.

This study also shows that many of the patients with nonschizophrenic psychotic disorders are also at increased risk of a second hospitalization if they do not take antipsychotics. This emphasizes the need for all patients with chronic psychotic disorders, not only those diagnosed with schizophrenia, to take antipsychotics.

Relevant to this topic, Bioque et al.^23^ included 119 patients in remission after a first episode of schizophrenia who were closely followed over 3 years. The study demonstrated that 22% of patients who finished the follow-up without relapsing were not taking any antipsychotic. In the data included in this current study, in the Swedish cohort, 21.4% of patients who finished the follow-up without relapsing were not taking any antipsychotic, and in the Israeli cohort, 34.6% of patients who finished the follow-up without relapsing did not take antipsychotics. Overall, 25.1% of patients who finished the follow-up without relapsing did not take antipsychotics in this current study, a finding very similar to Bioque et al.

Important findings were also noted by Mayoral-van Son et al.^24^ In a post hoc analysis of 209 patients after their first psychotic episode, individuals who stopped taking antipsychotics had higher relapse rates than patients who remained on a minimal dose of antipsychotics.

It is also interesting to note the differences in the compliance rates of antipsychotics and the rates of hospitalizations between the Israeli cohort and the Swedish cohort. The patients in the Swedish cohort after their first hospitalization tended to be more compliant with antipsychotics (86.4%), compared with the Israeli sample (64%). However, the overall risk of relapse in the Swedish sample (51.3%) and the Israeli sample (50.0%) was quite similar. Differences in compliance rates of psychotropics are often different in different countries.^25^

Limitations of this study include that this is not a randomized clinical trial and that the diagnoses in this study were clinical diagnoses extracted from electronic medical records only. However, the Clalit HMO database’s validity was previously displayed to be high.^26^ In addition, the same method assessing socioeconomic status was not used for the Swedish and the Israeli cohorts. Unfortunately, data on the education of an individual is not available for the Israeli cohort and neighborhood socioeconomic status is not available for the Swedish cohort. Another limitation is the fact that rehospitalization was defined as relapse in our study. These aspects do not always go together, since there are patients who are hospitalized for other causes, and there are patients who relapse who are not hospitalized. Other limitations include using filling of prescriptions from pharmacies as proxies for actual treatment, it is possible that patients withdrew medications but do not use it.

The study’s strength includes being based on data from 2 different continents and 2 different research groups, which increases the validity of the results.

## Conclusion

In summary, it was not possible to identify characteristics of patients who can safely discontinue antipsychotic medications and not later be hospitalized. Patients should be recommended to continue antipsychotic medications according to the current guidelines, especially male patients and those with narrowly defined schizophrenia diagnosis. Future research on this topic might attempt to identify biomarkers that accurately predict patient who can safely discontinue antipsychotics.

## Supplementary Material

sbaf011_suppl_Supplementary_Material

## Data Availability

Swedish data: The data used in this study cannot be made publicly available due to privacy regulations. According to the General Data Protection Regulation, the Swedish law SFS 2018:218, the Swedish Data Protection Act, the Swedish Ethical Review Act, and the Public Access to Information and Secrecy Act; these types of sensitive data can only be made available for specific purposes, including research, which meets the criteria for access to this sort of sensitive and confidential data as determined by a legal review. Readers may contact Professor Kristina Alexanderson (kristina.alexanderson@ki.se) regarding the data. Israeli data: Owing to Clalit Health Services’ data privacy regulations and per the institutional Helsinki and data utilization committee approvals for this study, the data used for this study cannot be shared.
