# Supplementary material for: Is it Possible To Identify Patients After Their First Hospitalization for a Psychotic Disorder Who Do Not Use Anti-Psychotics and are Not Later Rehospitalized?
Source: Schizophr Bull. 2025 Feb 21;52(1):sbaf011. doi: 10.1093/schbul/sbaf011 (PMC12809860; doi:10.1093/schbul/sbaf011)
Supplement: sbaf011_suppl_Supplementary_Material [file sbaf011_suppl_supplementary_material.docx]

**Supplementary Figure 1A.** Description of the study cohort formation in the Swedish data. Relapse refers to hospitalization due to psychotic disorder during follow-up*.

| 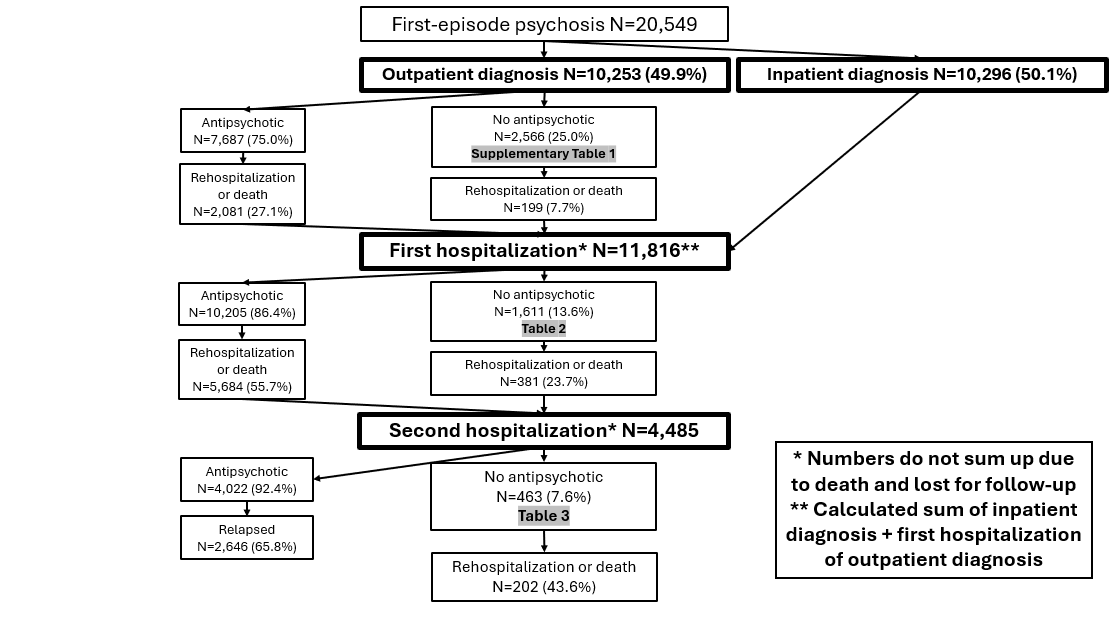  *Patients who were initially diagnosed as an outpatient, and then later been hospitalized only one time were not included in this study's analyses for risk of relapse.  **Supplementary Figure 1B.** Description of the study cohort formation in the Israeli data*.  **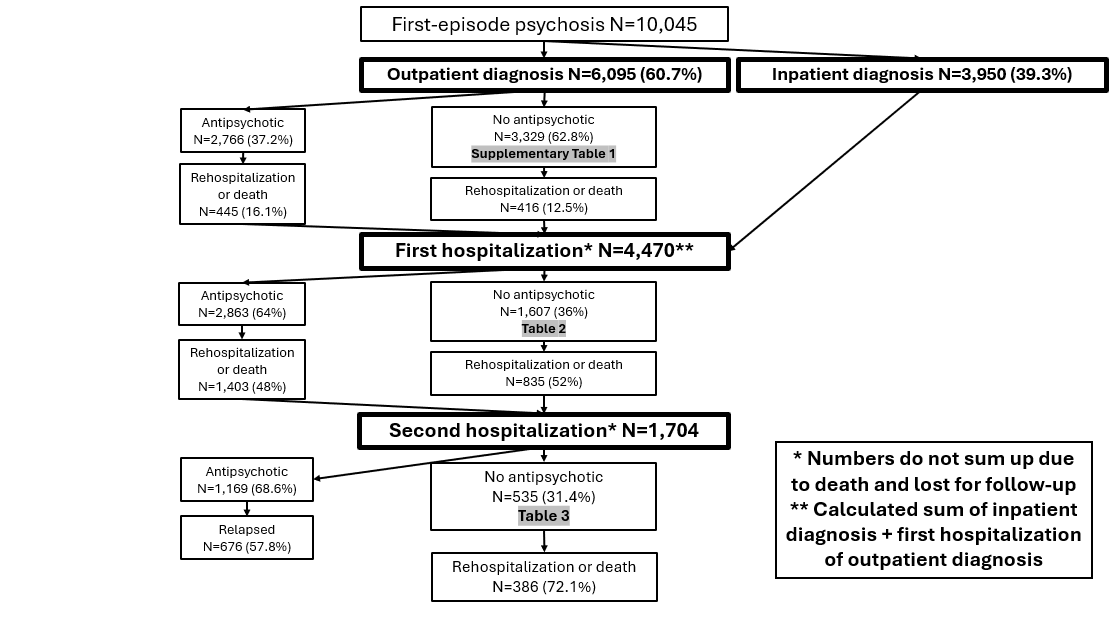**  *Patients who were initially diagnosed as an outpatient, and then later been hospitalized only one time were not included in this study's analyses for risk of relapse.  **Supplementary Table 1.** Risk of first hospitalization associated with sociodemographic and clinical characteristics in Israeli and Swedish cohorts of persons with first-episode schizophrenia-spectrum disorder diagnosed in outpatient care. | | | | | | | | |
| --- | --- | --- | --- | --- | --- | --- | --- | --- |
|  |  |  |  |  |  |  |  |  |
|  | **Israeli cohort** | |  |  | **Swedish cohort** | |  |  |
|  | **No relapse** | **Relapse** | **Crude** | **Multivariate** | **No relapse** | **Relapse** | **Crude** | **Multivariate** |
|  | **N=2913** | **N=416** | **HR (95%CI)** | **HR (95%CI)** | **N=2367** | **N=199** | **HR (95%CI)** | **HR 895%CI)** |
| Age, categorized, % (N) |  |  |  |  |  |  |  |  |
| <30 years | 87.82% (1695) | 12.18% (235) | 0.95 (0.78-1.15) | 1.09 (0.89-1.32) | 93% (1471) | 7% (111) | 0.79 (0.60-1.05) | 0.72 (0.54-0.97) |
| ≥30 years | 87.1% (1218) | 12.9% (181) | ref | ref | 91.1% (896) | 8.9% (88) | ref | ref |
| Male | 84.9% (1644) | 15.1% (293) | 1.78 (1.45-2.20) | 1.36 (1.10-1.69) | 90.7% (1474) | 9.3% (152) | 2.05 (1.48-2.85) | 1.70 (1.21-2.39) |
| Diagnosis type at diagnosis | |  |  |  |  |  |  |  |
| F20 Schizophrenia | 82.4% (1022) | 17.6% (219) | 1.97 (1.62-2.38) | 1.79 (1.47-2.18) | 95.7% (155) | 4.3% (7) | 0.48 (0.22-1.01) | 0.58 (0.27-1.25) |
| Other | 90.6% (1891) | 9.4% (197) | ref | ref | 92% (2212) | 8% (192) | ref | ref |
| Socioeconomic position |  |  |  |  |  |  |  |  |
| Low | 88.1% (751) | 11.9% (101) | 0.93 (0.74-1.16) | 0.87 (0.69-1.09) | 91.3% (1111) | 8.7% (106) | 1.24 (0.92-1.68) | 1.27 (0.93-1.73) |
| Medium | 86.3% (1602) | 13.7% (255) | ref | ref | 92.6% (862) | 7.4% (69) | ref | ref |
| High | 90.5% (332) | 9.5% (35) | 0.73 (0.52-1.03) | 0.84 (0.59-1.20) | 94.3% (394) | 5.7% (24) | 0.77 (0.49-1.23) | 0.92 (0.57-1.47) |
| No data | 90.1% (228) | 9.9% (25) |  |  |  |  |  |  |
| Substance use disorder | 67.2% (236) | 32.8% (115) | 3.65 (2.94-4.53) | 3.65 (2.94-4.53) | 87.5% (722) | 12.5% (103) | 2.62 (1.98-3.46) | 2.18 (1.62-2.94) |
| Benzodiazepine use | 85.8% (188) | 14.2% (31) | 1.17 (0.81-1.69) | 1.17 (0.80-1.70) | 91.3% (586) | 8.7% (56) | 1.23 (0.90-1.67) | 0.96 (0.67-1.36) |
| Antidepressant use | 92.5% (429) | 7.5% (35) | 0.54 (0.38-0.76) | 0.61 (0.42-0.86) | 91.5% (906) | 8.5% (84) | 1.22 (0.92-1.62) | 1.15 (0.84-1.58) |

**Supplementary Figure 2.** Meta-analysis results on the risk of first hospitalization associated with sociodemographic and clinical characteristics in Israeli and Swedish cohorts of persons with first-episode schizophrenia-spectrum disorder diagnosed in outpatient care.


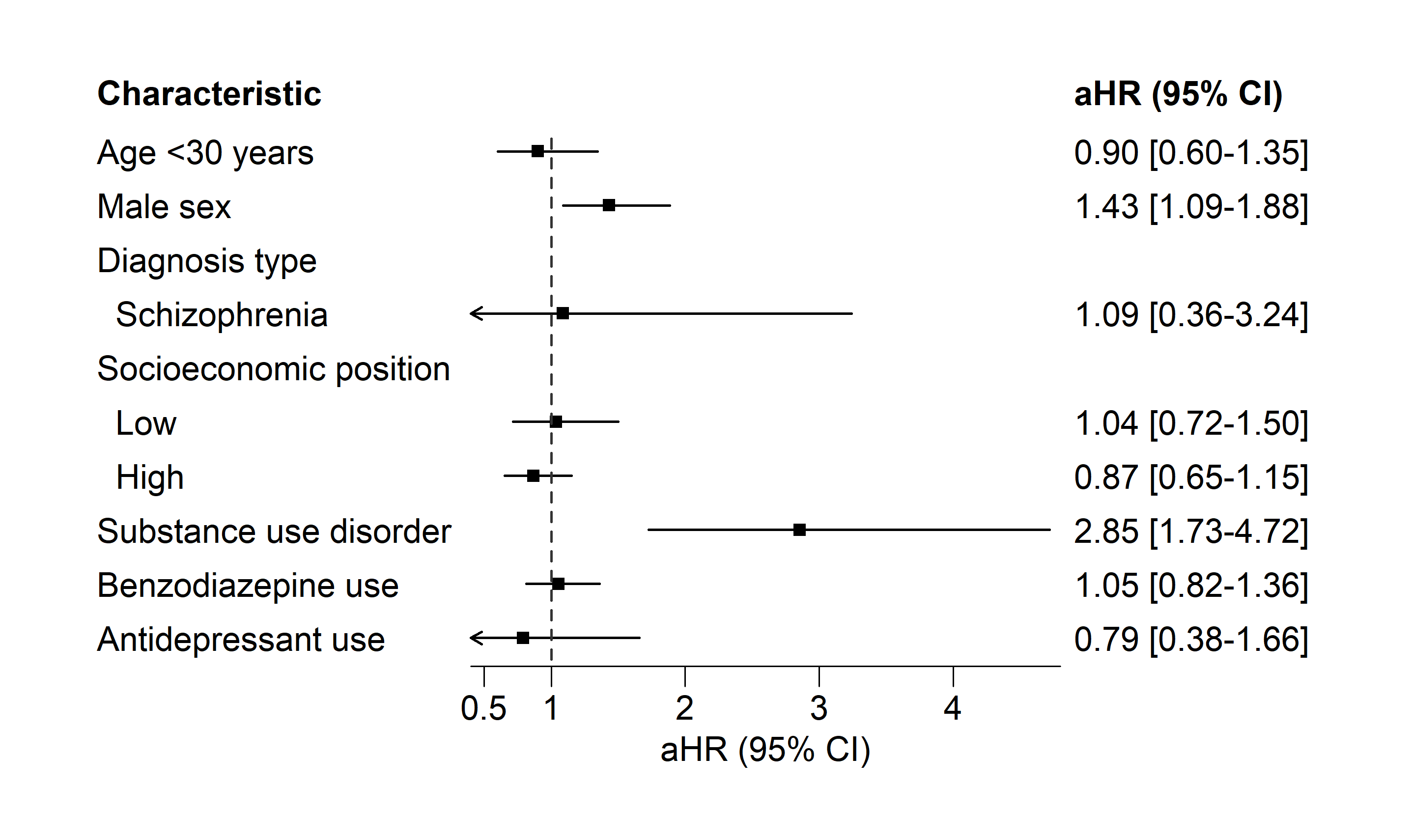


**Supplementary Table 2.** Description of the rates of relapse of patients with one hospitalization who did not take anti-psychotics among the different ICD-10 psychotic diagnoses

| **Diagnosis** | **Israeli cohort** | | | **Swedish cohort** | | |
| --- | --- | --- | --- | --- | --- | --- |
|  | **No relapse** | **Relapse** | **Total** | **No relapse** | **Relapse** | **Total** |
| **F20** | 42.5% (296) | 57.5% (400) | 696 | 56.9% (37) | 43.1% (28) | 65 |
| **F22** | 58.8% (10) | 41.2% (7) | 17 | 72.3% (81) | 27.7% (31) | 112 |
| **F23** | 51.7% (244) | 48.3% (228) | 472 | 80.1% (611) | 19.9% (152) | 763 |
| **F29** | 57.3% (173) | 42.7% (129) | 302 | 74.2% (445) | 24.8% (155) | 600 |
| **Other*** | 40.8% (49) | 59.2% (71) | 120 | 78.9% (56) | 21.1% (15) | 71 |
| **Total** | 48.0% (772) | 52.0% (835) | 1607 | 76.4% (1230) | 23.6% (381) | 1611 |

*: F21 (schizotypal disorder), F25 (schizoaffective disorder), F24 (induced delusional disorder), F28 (other non-organic psychotic disorder)

| **Supplementary Table 3.** Association between parental educational level and risk of first hospitalization in the main analysis of the Swedish cohort. | | | | |
| --- | --- | --- | --- | --- |
|  |  | **No relapse** | **Relapse** | **Crude** |
|  |  | **N=1230** | **N=381** | **HR (95%CI)** |
| Parental educational level | | | | |
| Unknown |  | 76.3 (338) | 23.7 (105) | 1.06 (0.81-1.38) |
| Low |  | 72.1 (147) | 27.9 (57) | 1.26 (0.92-1.73) |
| Medium |  | 77.1 (400) | 22.9 (119) | Ref |
| High |  | 77.5 (345) | 22.5 (100) | 0.99 (0.76-1.30) |
